# Maternal smoking around birth and its influence on offspring allergic diseases: A mendelian randomization study

**DOI:** 10.1016/j.waojou.2024.100875

**Published:** 2024-02-04

**Authors:** Qiqi Ruan, Yu Jiang, Yuan Shi

**Affiliations:** a Department of Neonatology, Children's Hospital of Chongqing Medical University, National Clinical Research Center for Child Health and Disorders, Ministry of Education Key Laboratory of Child Development and Disorders, Chongqing Key Laboratory of Child Rare Diseases in Infection and Immunity, Chongqing, People's Republic of China; b Department of Pediatrics, Women and Children's Hospital of Chongqing Medical University, Chongqing, People's Republic of China

**Keywords:** Mendelian randomization, Maternal smoking around birth, Breastfeeding, Allergic diseases

## Abstract

**Objective:**

The influence of maternal smoking around birth (MSAB) on offspring allergic diseases, specifically childhood asthma (CA), allergic rhinitis (AR), allergic conjunctivitis (AC), and atopic dermatitis (AD) remains incompletely understood. We performed a rigorous mendelian randomization (MR) study to obtain the unconfounded association between MSAB and allergic diseases in offspring with and without adjustment for the effect of breastfeeding.

**Methods:**

Utilizing publicly available information of MSAB, breastfeeding, CA, AR, AC, and AD from large-scale genome-wide association studies (GWAS), we performed a two-sample mendelian randomization (TSMR) analysis to assess the respective causal relationship of MSAB and breastfeeding to allergic diseases in offspring. To get a reliable conclusion, MR Egger regression, weighted median, and inverse variance weighted (IVW) were employed to estimate the causality, with IVW as the primary analysis. Multivariate MR (MVMR) analysis was used to assess the effect of MSAB on allergic diseases after adjusting for breastfeeding's impact. Sensitivity analysis was conducted using the Cochran Q test, MR-Egger, and leave-one-out approaches to ensure the reliability and stability of results.

**Results:**

The TSMR analysis demonstrated MSAB increased the risks of CA (P_IVW_ = 0.013, OR: 1.018, 95%CI: 1.004 to 1.033) and AD (P_IVW_ = 0.006, OR: 8.293, 95%CI: 1.815 to 37.884) in offspring. Conversely, breastfeeding decreased the risk of CA (P_IVW_ <0.001, OR: 0.946, 95%CI: 0.918 to 0.974). MSAB still increased the risks of CA (P = 0.0497, OR: 1.013, 95%CI: 1.000017 to 1.026) and AD (P = 0.003, OR: 13.800, 95%CI: 2.490 to 269.246) after adjusting for breastfeeding. We observed no strong indication of a negative causality between MSAB and AC and AR.

**Conclusion:**

Our findings provided robust evidence of the adverse effects of MSAB on offspring. We emphasized the urgency of smoking cessation around birth and the importance of breastfeeding even in smoking mothers.

## Introduction

Numerous maternal exposures significantly contribute to the health problems of offspring.^1^ The estimated prevalence of maternal smoking during pregnancy^2^ was about 10% and even higher due to under-reporting in many countries and regions. This widespread habit could impair the intrauterine environment and adversely affect the developing organs of offspring.^3^^,^^4^ Previous studies have extensively documented the correlation between maternal smoking during pregnancy and low birth weight,^5^, ^6^, ^7^ premature births,^8^ congenital heart defects,^9^^,^^10^ chronic obstructive pulmonary disease,^11^ anxiety and depression,^12^ and other disorders in offspring.

The prevalence of allergic diseases^13^^,^^14^ has been steadily rising, significantly affecting people's quality of life and emerging as a critical public health concern. Despite considerable attention, the specific impact of maternal smoking around birth (MSAB) on allergic diseases in offspring remains unclear. Several studies have investigated the effects of maternal smoking during pregnancy on wheezing and childhood asthma (CA)^15^^,^^16^ in offspring. According to a systematic review and meta-analysis, there was a strong association between prenatal maternal smoking and asthma in children ≤2 years.^17^ Although this association weakened with age, it remained statistically significant. Prenatal smoke exposure has also been reported as a risk factor for allergic rhinitis (AR),^18^ allergic conjunctivitis (AC),^19^ and atopic dermatitis (AD)^20^ in the offspring, but the evidence is still mixed.^21^^,^^22^

Numerous studies have proven that breastfeeding^23^ has a wide range of health benefits for both mothers and children, appearing as a protective factor^24^ against allergic diseases. Thus far, conclusive results regarding whether breastfeeding reduces the risk of allergic diseases in offspring induced by MSAB are still lacking.

Remarkably, confirming causal associations in observational studies is inherently challenging because of limited sample sizes, confounding factors,^25^ and reverse causality.^26^ To overcome these limitations, the mendelian randomization (MR) study takes advantage of randomly assigned single-nucleotide polymorphisms (SNPs) strongly associated with the exposure of interest. These SNPs are analyzed as instrumental variables (IVs) so that the study could be less susceptible to confounding factors such as subsequent environmental, socioeconomic, and behavioral factors.^27^ Furthermore, it could facilitate deeper research using ample public information.

To our knowledge, this is the first MR study to assess the effects of MSAB on allergic diseases in offspring with and without adjustment of the effect of breastfeeding. Employing a two-sample mendelian randomization (TSMR) analysis, we explored the respective causal relationship of MSAB and breastfeeding to allergic diseases in offspring. The multivariate MR (MVMR) analysis was used to assess the effect of MSAB on allergic diseases after adjusting for the effect of breastfeeding. Our aim was to shed light on the detrimental effects of MSAB and offer robust recommendations for public health initiatives.

## Methods

### Ethical approval

Ethics approval was not required for this MR study because it is based solely on publicly available genome-wide association studies (GWAS) data without the direct engagement of participants.

### Exposures

Maternal smoking status was identified by participants answering “Did your mother smoke regularly around the time when you were born?” and breastfeeding status was confirmed by answering “Were you breastfed when you were a baby?” with response options of “Yes”,” No”, “Do not know”, “Prefer not to answer”. There were no detailed measures of specific doses and duration of breastfeeding or maternal smoking.

### Data resources

Genetic associations with the exposures and outcomes of interest were identified using publicly available GWAS. Independent instrumental SNPs for MSAB (https://gwas.mrcieu.ac.uk/datasets/ukb-b-17685/, n = 397 732), breastfed as a baby(https://gwas.mrcieu.ac.uk/datasets/ukb-b-13423/, n = 352 094) and CA (https://gwas.mrcieu.ac.uk/datasets/ukb-d-ASTHMA_CHILD/, n = 361 194) in people of European ancestry were obtained from publicly GWAS summary dataset from the UK Biobank (UKB). While AR (https://gwas.mrcieu.ac.uk/datasets/finn-b-ALLERG_RHINITIS/, n = 217 914), AC (https://gwas.mrcieu.ac.uk/datasets/finn-b-H7_ALLERGICCONJUNCTIVITIS/, n = 218 792), AD (https://gwas.mrcieu.ac.uk/datasets/finn-b-L12_ATOPIC/, n = 205 764) were from Finnish Biobank. Finnish Biobank^28^ and UKB^29^ are both large-scale biomedical databases and research resources, containing genetic information and health data for approximately 500,000 Finnish and 500 000 UK participants, respectively, and contributing to the studies and discoveries that improve human health.

### Instrumental single-nucleotide polymorphisms

We took a series of quality control steps to select appropriate instrumental single-nucleotide polymorphisms (SNPs).^30^ As shown in Fig. 1a, the instrumental SNPs for the exposure of interest were identified based on 3 assumptions. Thus, first, we selected independent SNPs in the GWAS of MSAB and breastfeeding with the lowest P-value threshold (*P* < 5e-8) and r^2^ < 0.001, window size = 10 000 kb to guarantee that SNPs were strongly associated with exposures and remove linkage disequilibrium. Second, as a result of phenome-wide scanning (http://www.phenoscanner.medschl.cam.ac.uk/), we excluded SNPs associated with confounder traits. Third, we further excluded SNPs related to the outcomes (*P* < 5e-8).

**Fig. 1a:**
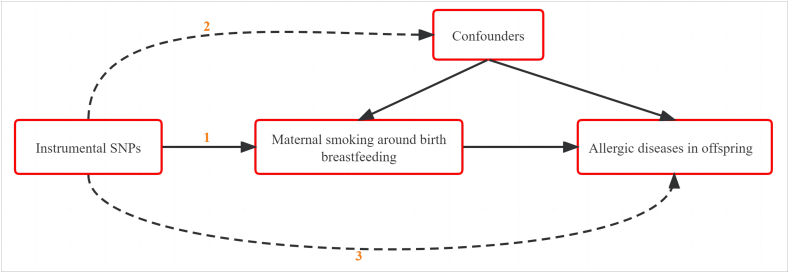
The assumptions that the instrumental SNPs for the exposure of interest must satisfy: 1) The selected genetic instrumental SNPs must be strongly associated with the exposure of interest, 2) the SNPs should not be associated with confounders of exposure and outcomes, and 3) the SNPs should affect the outcomes only via the exposure.

### Statistical analysis

We conducted the following robust MR methods to estimate the robust causality of MSAB and breastfeeding on offspring allergic diseases: MR Egger regression, weighted median, inverse variance weighted (IVW). These classic analytical methods gave reliable integration results based on different assumptions of TSMR analysis.^31^ As the primary analysis, we mainly endorsed the results of IVW.^32^ MVMR analysis was used to adjust for the effect of breastfeeding.

Sensitivity analyses were performed using the MR-Egger intercept test for horizontal pleiotropy, the Cochran Q test and MR-Egger regression for heterogeneity, and the leave-one-out MR approaches to ensure the reliability and stability of the relationship between SNPs and exposures. We also performed MR analyses of scatter plots, forest plots, and funnel plots to directly show the relationship of MSAB and breastfeeding to outcomes of interest.

We used a *P*-value<0.05 as the threshold for statistical significance. All the MR analysis was implemented in the TwoSampleMR package^33^ (version 0.5.6) in R (version 4.2.2).

## Results

Fig. 1b illustrated the flow chart of this study. We finally utilized 13 SNPs of MSAB and 4 SNPs of breastfeeding from the UKB which met the 3 assumptions selecting instrumental predictors, while the SNPs rs7899608, rs2183947, rs2428019 of MSAB, and rs9925536 and rs1567820 of breastfeeding were excluded for being the outliers and associated with the confounder risks. Detailed information of SNPs for exposures was shown in Table 1.

**Fig. 1b:**
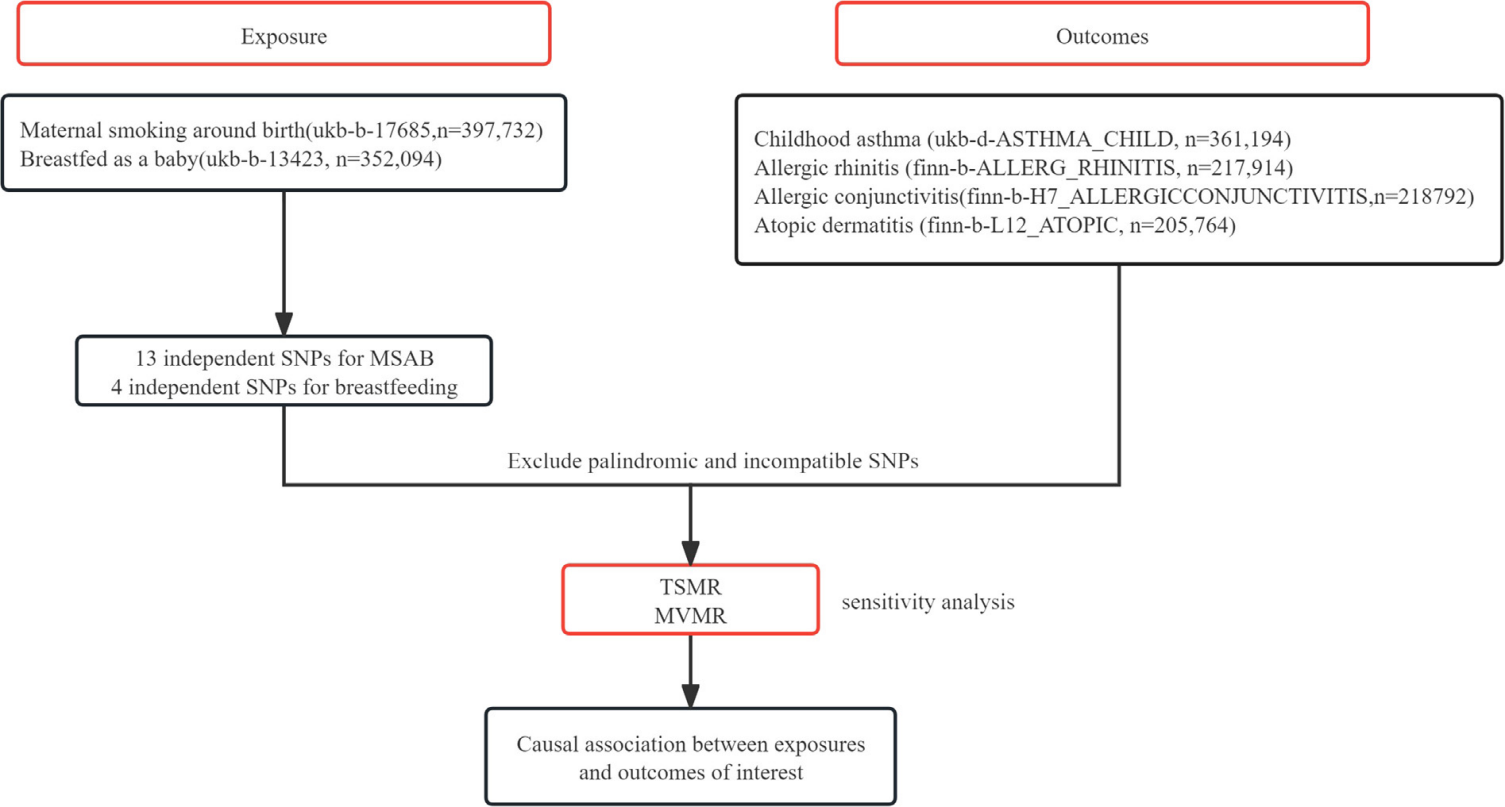
The analysis pipeline of the study

**Table 1:** Detailed information of SNPs in this MR study.

Exposure	ID. exposure	Sample size	Chr	Position	SNPs	EA	NEA	se	β	EAF	*P-value*
Maternal Smoking around Birth	ukb-b-17685	397732	1	44097438	rs12405972	T	G	0.0011	−0.0081	0.3483	6.40E-14
			2	164928199	rs35566160	G	A	0.0012	0.0064	0.2748	4.50E-08
			4	140939110	rs36072649	A	T	0.0011	−0.0072	0.3808	1.10E-11
			5	50748173	rs4865667	T	C	0.0011	−0.0058	0.3878	3.40E-08
			7	32315613	rs10226228	G	A	0.0011	0.0074	0.3702	4.00E-12
			7	114951541	rs62477310	C	T	0.001	−0.0058	0.4867	2.00E-08
			7	75196531	rs794356	A	G	0.0011	−0.0061	0.4421	1.90E-08
			8	93114414	rs7002049	C	T	0.0012	0.0076	0.7847	1.40E-09
			9	136468701	rs75596189	T	C	0.0016	0.0121	0.11	2.10E-13
			9	14453010	rs1323341	G	A	0.0012	−0.0068	0.7816	3.90E-08
			15	78870803	rs576982	T	C	0.0012	−0.0093	0.2279	2.30E-14
			16	24798079	rs12923476	A	G	0.0012	−0.0069	0.2569	4.80E-09
			20	61984317	rs6011779	T	C	0.0013	−0.0099	0.8087	2.50E-14
Breastfed as a baby	ukb-b-13423	352094	6	85370492	rs9362076	G	A	0.0012	−0.0068	0.7275	1.300E-08
			6	31060219	rs2535296	G	A	0.0014	−0.0079	0.1842	9.100E-09
			7	132691858	rs56182580	C	T	0.0012	−0.0068	0.2813	1.300E-08
			14	42499198	rs8010613	T	C	0.0012	−0.0065	0.3060	2.400E-08

Abbreviations: SNPs, single-nucleotide polymorphisms; MR, mendelian randomization; Chr, chromosome; EA, effect allele; EAF, effect allele frequency

As shown in Supplementary Table 1, the Cochran Q test and MR–Egger heterogeneity analysis showed little influence in the causal effect estimate for MSAB and breastfeeding on the outcomes with all SNPs included in the model, except for allergic rhinitis (*P*_Cochran Q test_ = 0.03, *P*_MR_ egger = 0.07). In addition, we did not find any evidence of horizontal pleiotropy for MSAB and breastfeeding on any outcomes (with all *P* values larger than 0.05). No potentially influential SNP was identified in the “leave-one-out” sensitivity analysis (Plots were shown in Supplementary Fig 1), demonstrating that the conclusion was stable. Supplementary Fig 2.1-2.3 presented scatter plots, forest plots, and funnel plots illustrating the estimated effect sizes of these SNPs on the outcomes.

TSMR analysis (Table 2) showed the causal effect estimates assessed by different MR methods. We observed that genetically predicted MSAB was significantly associated with the risk of CA (P_IVW_ = 0.013, OR: 1.018, 95%CI: 1.004 to 1.033) and AD (P_IVW_ = 0.006, OR: 8.293, 95%CI: 1.815 to 37.884) in offspring, but no strong indication of a negative causality with AR and AC. Breastfeeding decreased the risk of CA (P_IVW_ <0.001, OR: 0.946, 95%CI: 0.918 to 0.974), but did not show significant protection against AR, AC and AD.

**Table 2:** MR assessment of the respective causal relationship of MSAB and breastfeeding to allergic diseases in offspring.

Exposure	Outcome	method	NSNPs	β	OR (95%CI)	*P*
MSAB	CA	MR Egger	13	0.033	1.033(0.961, 1.111)	0.393
		Weighted median	13	0.021	1.021(1.001, 1.041)	0.038
		IVW	13	0.018	1.018(1.004, 1.033)	0.013
	AR	MR Egger	13	8.231	3755.339(0.473, 2.982e+7)	0.100
		Weighted median	13	0.064	1.067(0.096, 11.820)	0.958
		IVW	13	0.638	1.893(0.360, 9.952)	0.451
	AC	MR Egger	13	−2.609	0.074(6.901e-5,78.478)	0.479
		Weighted median	13	−0.642	0.526(0.096, 2.885)	0.459
		IVW	13	−0.846	0.429(0.119,1.558)	0.199
	AD	MR Egger	13	−0.854	0.426(0.000, 1593.654)	0.842
		Weighted median	13	2.425	11.304(1.323, 96.612)	0.027
		IVW	13	2.115	8.293(1.815, 37.884)	0.006
Breastfeeding	CA	MR Egger	4	−0.415	0.660(0.449,0.971)	0.169
		Weighted median	4	−0.047	0.954(0.920,0.990)	0.013
		IVW	4	−0.056	0.946(0.918,0.974)	<0.001
	AR	MR Egger	4	−52.994	9.661e-24(3.653e-63,2.555e+16)	0.371
		Weighted median	4	4.521	91.885(1.296,6.514e+3)	0.038
		IVW	4	1.945	6.991(1.744e-2,2.802e+3)	0.525
	AC	MR Egger	4	−18.078	1.409e-8(1.696e-27,1.171e+11)	0.501
		Weighted median	4	1.196	3.308(0.123,89.142)	0.476
		IVW	4	0.449	1.566(0.106,23.243)	0.744
	AD	MR Egger	4	25.845	1.675e+11(1.080e-17,2.602e+39)	0.517
		Weighted median	4	−1.590	0.204(4.187e-3,9.935)	0.423
		IVW	4	−1.960	0.141(3.085,6.429)	0.315

Abbreviations: MR: mendelian randomization; SNPs, single-nucleotide polymorphisms; OR, odds ratio; CI, confidence interval; CA, childhood asthma; AR, allergic rhinitis; AC, allergic conjunctivitis; AD, atopic dermatitis; IVW: inverse-variance weighted

**Fig. 2:**
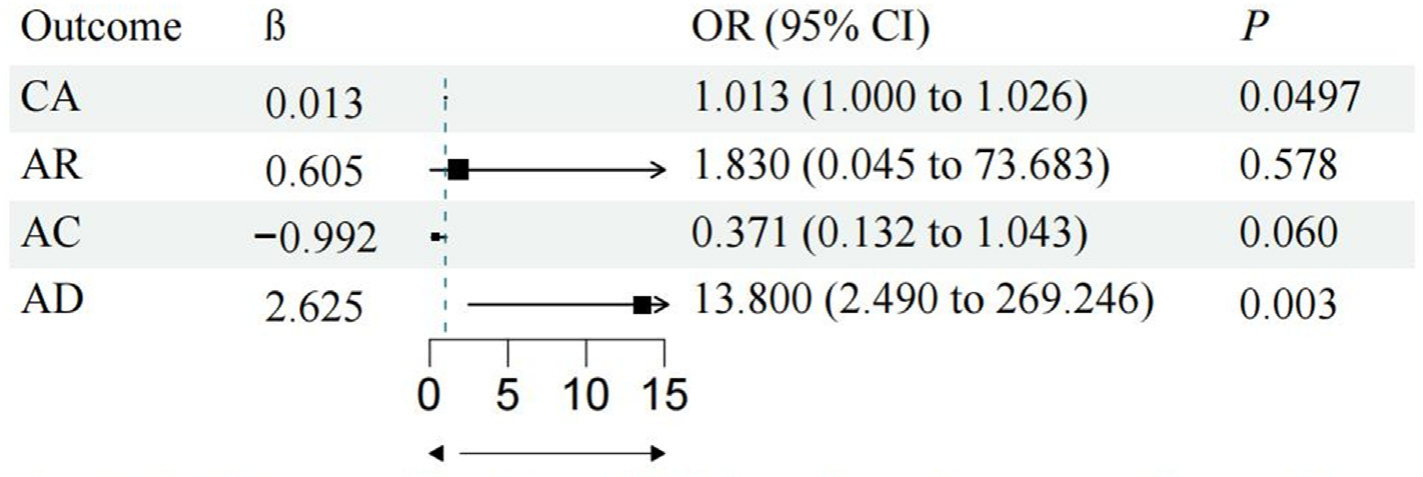
MVMR assessed the effect of MSAB on allergic diseases in offspring after adjusting for the effect of breastfeeding

In the MVMR analysis (Fig. 2), MSAB still increased the risks of CA (P = 0.0497, OR: 1.013, 95%CI: 1.000017 to 1.026) and AD (P = 0.003, OR: 13.800, 95%CI: 2.490 to 269.246) after adjusting for the effect of breastfeeding.

## Discussion

Through our meticulous MR study, we discovered that MSAB increased the risks of CA and AD in offspring, regardless of adjustments made for the effect of breastfeeding. However, our analysis did not reveal substantial evidence of a causal effect on AR and AC. These findings filled a gap in previous research and may contribute to a better understanding of the effects of maternal smoking and breastfeeding on offspring.

Despite its well-known health risks, smoking during pregnancy is still prevalent in many countries, with the highest estimated prevalence in the European region. However, there are limited studies revealed the actual effects of maternal smoking on offspring health because the findings of observational studies and related secondary studies may be influenced by confounding factors and reverse causality.^26^ Fortunately, the MR study is to utilize publicly available information from the large-scale GWAS to explore the reliable correlation between a genetically determined "exposure" and an "outcome" and to eliminate the inherent limitations associated with observational studies.

Consistent with our findings, a previous systematic review and meta-analysis showed that prenatal maternal smoking contributed to childhood asthma and wheezing.^34^ Mahon et al^35^ also reported that grandmothers with a history of smoking during pregnancy were related to a higher asthma risk (OR [95% CI]: 1.49[1.06–2.11]) and worse lung function (β [95%CI]: 1.04[−1.9 to −0.16]) in male grandchildren, highlighting the entrenched effects of smoking across generations. Even though Kantor et al^36^ reported no significant association between maternal smoking during pregnancy and the incidence of AD, our study suggested that MSAB increased the risks of AD. In addition, the relationship between maternal smoking and AR was not statistically significant and somewhat inconsistent with many published studies,^37^ which may be because of confounding factors such as education level, socioeconomic status, life habits, and selection bias.

Previous studies have shown that breastfeeding may improve immune function, thereby providing short- and long-term protection to offspring against infection and allergies.^38^^,^^39^ A prospective cohort study reported that infants who were breastfed were less likely to develop asthma^40^ and other childhood morbidities, demonstrating the substantial health and economic implications of breastfeeding. But Lodge^38^ found a limited association between breastfeeding and a reduced risk of AR and eczema in the first 2 or 5 years of life, which were the same as our findings. Although our study showed that breastfeeding did not significantly diminish the negative effects of MSAB on CA and AD in offspring, the P-value for MSAB increased CA risk was significantly higher, extremely close to 0.5 (0.0497), which was statistically marginal significance. GWAS data are limited currently, and we do not have a systematic understanding of the protective effects of breastfeeding against other diseases in offspring by MR study, but it is obvious that maternal smoking without breastfeeding is even worse.

Under the assumptions of MR, our findings suggested that MSAB increased the risks of CA and AD in their offspring. However, the underlying mechanisms for the causal effects remain unclear. Smoking during maternal pregnancy means putting the fetus at risk of second-hand smoke exposure during a critical period of organ development. Oocytes and embryos are particularly susceptible to tobacco compounds,^41^ leading to delayed embryonic morphogenesis. Tobacco components and combustion products can reach babies either through breast milk or inhalation after birth. Previous research has suggested that maternal smoking leads to telomere shortening in offspring.^42^ It is worth mentioning that MSAB may also induce transgenerational epigenetic modifications, such as changes in fetal T cell function and DNA methylation. All these factors alter the adaptive and innate immune systems of newborns. Furthermore, it is possible that airway remodeling could contribute to CA, while impaired immune responses could play a role in DA. Further studies are essential to explore the role of maternal smoking in the development of these allergic diseases.

The strength of this study was that we selected SNPs in UKB strongly associated with MSAB and breastfeeding as IVs, indicating that the IVs were robust enough to draw our conclusions. In addition, we applied MVMR to strengthen the effect estimates, making the findings more reliable. The limitations were as follows. Firstly, the summary GWAS data we extracted consisted merely of individuals of European descent and there may be sample overlap. Therefore, we should be cautious in extending our findings to other racial and ethnic groups. Secondly. heterogeneity was detected in the analysis regarding AR, which may be associated with gender, age, etc. However, as the primary method, IVW analysis provided a reliable conclusion. Thirdly, our results from the MR analysis might be biased by some potential pleiotropy. Nevertheless, sensitivity analysis did not reveal significant confounding effects. Fourthly, the GWAS data we applied on MSAB and breastfeeding, unfortunately, did not show the specific duration and dose of maternal smoking and breastfeeding, but we still drew a relatively reliable conclusion by conducting MR analysis.

## Conclusion

In conclusion, MR analysis is a robust method for epidemiological studies, and our results demonstrated an increased risk of CA and AD in her offspring induced by MSAB. We emphasized the necessity of maternal smoking cessation around birth and encouraged breastfeeding even if mothers smoked, which could have implications for public health interventions.

## Abbreviations

AC, allergic conjunctivitis; AR, allergic rhinitis; AD, atopic dermatitis; Chr, chromosome; CA, childhood asthma; CI, confidence interval; EA, effect allele; EAF, effect allele frequency; GWAS, genome-wide association studies; IVs, instrumental variables; IVW, inverse variance weighted; MVMR, multivariate mendelian randomization; NA, not applicable; OR, odds ratio; RCT, randomized controlled studies; SNPs, single-nucleotide polymorphisms; TSMR, two-sample mendelian randomization; UKB, UK Biobank.

## Funding source

None.

## Availability of data and materials

The relevant data for this study can be found at https://gwas.mrcieu.ac.uk/: 1. maternal smoking around birth (https://gwas.mrcieu.ac.uk/datasets/ukb-b-17685/), 2. breastfed as a baby(https://gwas.mrcieu.ac.uk/datasets/ukb-b-13423/), 3. childhood asthma (age<16) (https://gwas.mrcieu.ac.uk/datasets/ukb-d-ASTHMA_CHILD/), 4. allergic rhinitis (https://gwas.mrcieu.ac.uk/datasets/finn-b-ALLERG_RHINITIS/), 5. allergic conjunctivitis(https://gwas.mrcieu.ac.uk/datasets/finn-b-H7_ALLERGICCONJUNCTIVITIS/), 6. atopic dermatitis (https://gwas.mrcieu.ac.uk/datasets/finn-b-L12_ATOPIC/).

## Author contributions

QR designed the study, conducted all the analysis and wrote the first draft of the manuscript. YJ interpreted the results of the data analyses and edited the manuscript. YS helped to interpret the results and edited the manuscript. All authors revised the manuscript and approved the final manuscript.

## Ethics approval

Ethical approval was not required for this MR study because it is based solely on publicly available genome-wide association studies (GWAS) data without the direct engagement of participants.

## Authors’ consent for publication

All authors of the manuscript titled “Maternal Smoking around Birth and Its Influence on Offspring Allergic Diseases: A Mendelian Randomization Study” agree to submit it for publication in the World Allergy Organization Journal. We hereby grant editors the right to edit the manuscript and publish it. We confirm that this manuscript is an original work, has not been published before, and is not under consideration for publication elsewhere. We further confirm that all authors have contributed significantly to the conception, design, analysis, and interpretation of the data, and have participated in drafting and revising the manuscript. We have reviewed the final version of the manuscript and approve it for publication.

## Declaration of competing interest

The authors declare that the research was conducted in the absence of any commercial or financial relationships that could be construed as a potential conflict of interest.
