# Supplementary material for: Maternal smoking around birth and its influence on offspring allergic diseases: A mendelian randomization study
Source: World Allergy Organ J. 2024 Feb 4;17(2):100875. doi: 10.1016/j.waojou.2024.100875 (PMC10862070; doi:10.1016/j.waojou.2024.100875)
Supplement: Multimedia component 1 [file mmc1.docx]

Supplementary Material

**Maternal Smoking around Birth and Its Influence on Offspring Allergic Diseases: A Mendelian Randomization Study**

# Supplementary Figures and Tables

## Supplementary Tables

Supplementary Table 1. Heterogeneity and horizontal pleiotropy analysis for MSAB and breastfeeding on the outcomes.

|  | Outcomes | Heterogeneity analysis | | Horizontal pleiotropy analysis |
| --- | --- | --- | --- | --- |
|  |  | *P*-value  (MR–Egger) | *P*-value  (Cochran Q test) | *P*-value  (MR–Egger) |
| MSAB | CA | 0.40 | 0.46 | 0.69 |
|  | AR | 0.72 | 0.55 | 0.12 |
|  | AC | 0.97 | 0.97 | 0.63 |
|  | AD | 0.62 | 0.66 | 0.49 |
| Breastfeeding | CA | 1.00 | 0.34 | 0.21 |
|  | AR | 0.07 | 0.03 | 0.36 |
|  | AC | 0.55 | 0.59 | 0.49 |
|  | AD | 0.20 | 0.22 | 0.49 |

## Abbreviations: MSAB, maternal smoking around birth; CA, childhood asthma; AR, allergic rhinitis; AC, allergic conjunctivitis.

## Supplementary Figures


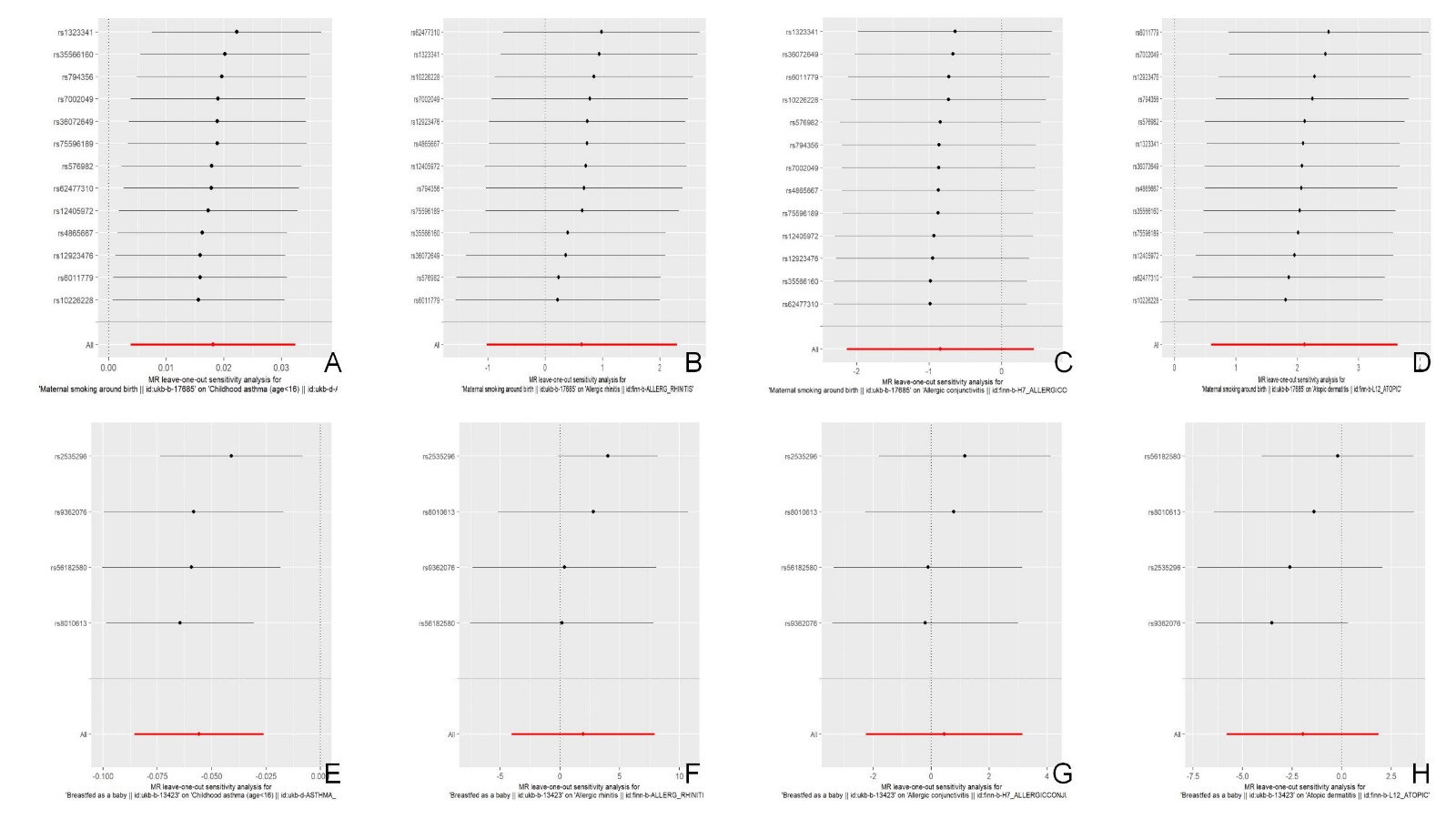


**Supplementary Fig 1** Leave-one-out MR analysis for MSAB and breastfeeding on the outcomes. A. MSAB-CA; B. MSAB-AR; C. MSAB-AC; D. MSAB-AD; E. breastfeeding-CA; F. breastfeeding-AR; G. breastfeeding-AC; H. breastfeeding-AD.


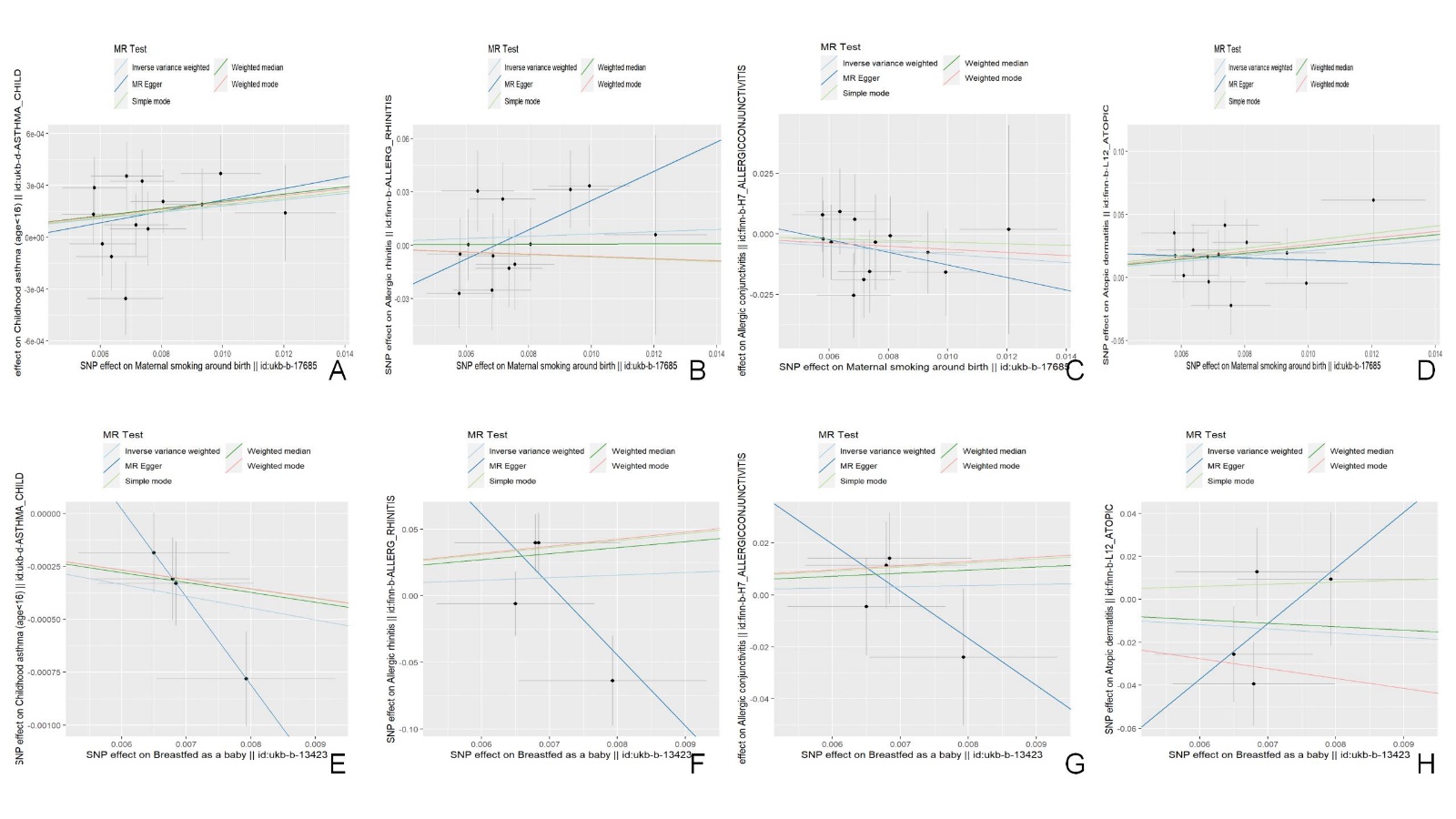


**Supplementary Fig 2.1** Scatter plots for MSAB and breastfeeding on the outcomes. A. MSAB-CA; B. MSAB-AR; C. MSAB-AC; D. MSAB-AD; E. breastfeeding-CA; F. breastfeeding-AR; G. breastfeeding-AC; H. breastfeeding-AD. Analysis was conducted using the conventional IVW, Weighted median, MR-Egger, weighted mode, and simple mode methods.


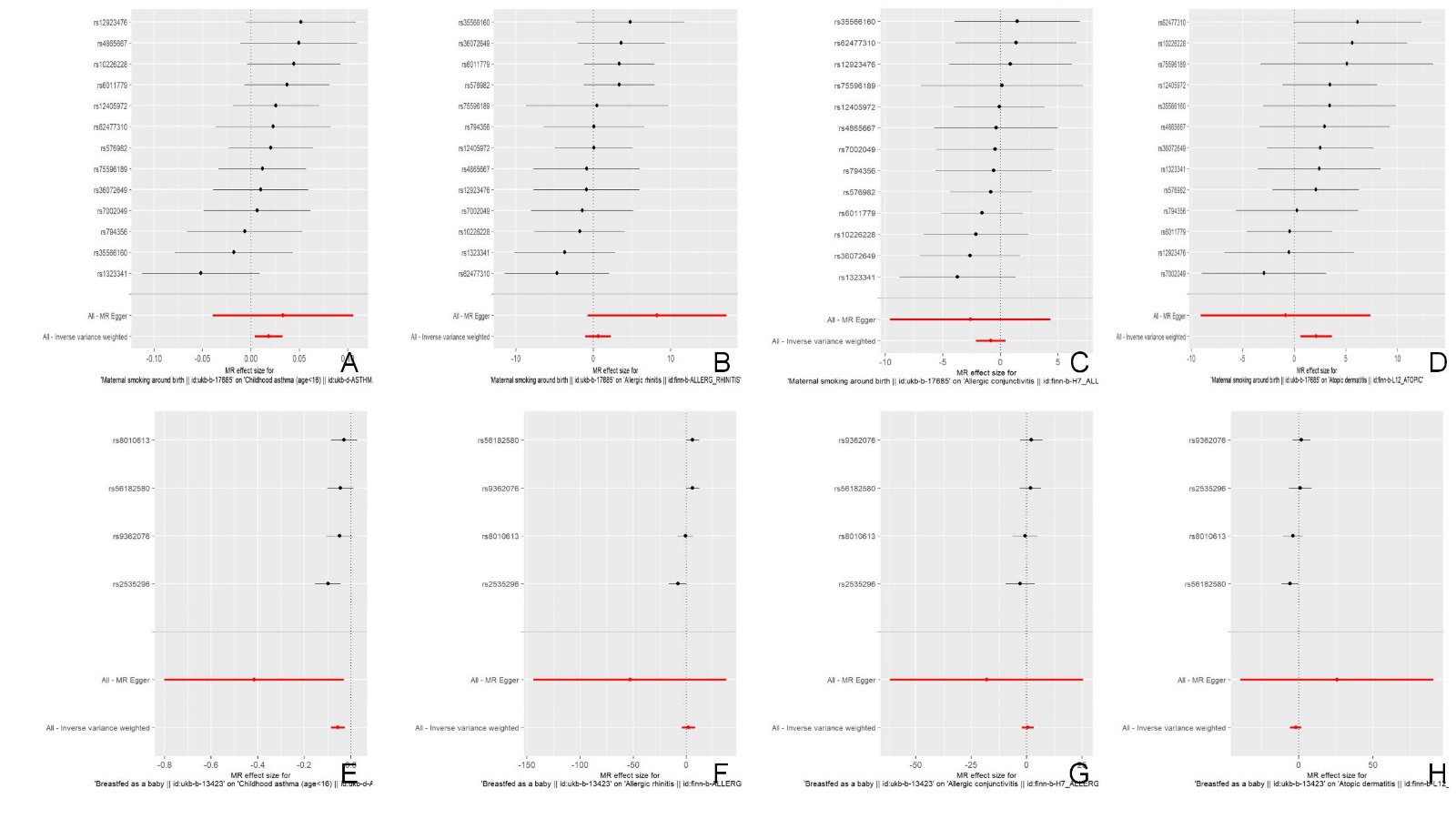
**Supplementary Fig 2.2** Forest plots for MSAB and breastfeeding on the outcomes. A. MSAB-CA; B. MSAB-AR; C. MSAB-AC; D. MSAB-AD; E. breastfeeding-CA; F. breastfeeding-AR; G. breastfeeding-AC; H. breastfeeding-AD. Analysis was conducted using the conventional IVW and MR-Egger methods.


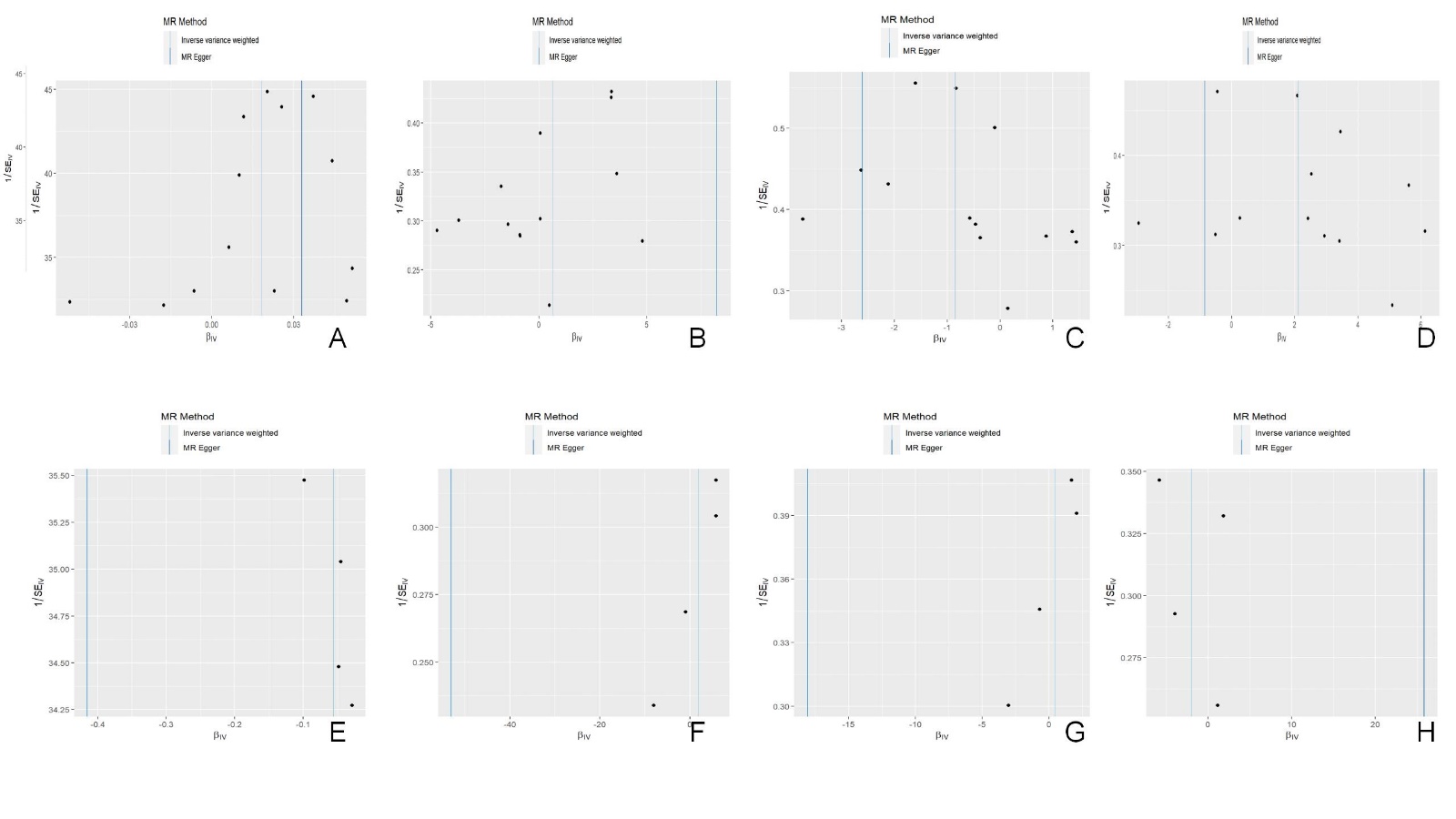


**Supplementary Fig 2.3.** funnel plots for MSAB and breastfeeding on the outcomes. A. MSAB-CA; B. MSAB-AR; C. MSAB-AC; D. MSAB-AD; E. breastfeeding-CA; F. breastfeeding-AR; G. breastfeeding-AC; H. breastfeeding-AD. Analysis was conducted using the conventional IVW and MR-Egger methods.
